# The Influence the Dental Profession Sustains toward Civilization

**Published:** 1913-10-15

**Authors:** L. J. McRae

**Affiliations:** Memphis


### THE INFLUENCE THE DENTAL PROFESSION SUSTAINS TOWARD CIVILIZATION.

BY DR. L. J. MCRAE, MEMPHIS.

Read before the Tennessee State Dental Association, June 5, 1913.

"Full many a gem of purest ray serene,
The dark unfathomed caves of Ocean bear,
Full many a flower is born to blush unseen,
And waste its sweetness on the desert air."

We all recognize the fact that through the flight of these many ages from the darkness of ignorance toward the light of civilization, that the conditions set forth in these poetic lines have been and are still sadly true, and even in this early dawn of the Twentieth Century, we can still hear the distress call from humanity, floating in the very atmosphere in which we exist, calling us to fathom the now unknown deep and rescue the precious gems, or to make fertile the now desert land and save the fragrance of the ever-blooming and wasting flowers. Thus the compass of civilization is directing; thus all humanitarians follow as it leads them. It is my earnest desire to show briefly that there is no greater humanitarian force at work to-day in the world than the Dental Profession; that no other field offers itself as a greater factor in raising the standards and shaping the future of modern civilization than the Dental Profession of to-day. My first effort will be to show our relationship to civilization and to present the influence which our special field—the mouth—sustains toward the body in general.

The mouth is to the body as the picket soldiers are to the army as it slumbers around bivouac fires on martial soil of the enemies' stronghold. For the picket force to neglect this important post, whose duty was assigned by the Creator, is to expose the entire army, the body, to its merciless foes, many of which so often give no quarter, save in death. The thirty-two feet of digestive tract is either the seat of or sustains important relation by direct or indirect connection with most diseases to which the body is subject. No part of this tract is so conspicuous in health or disease as the first two or three inches called the mouth, as here are found the teeth, the tongue, the cheeks and the saliva, with the proper heat and moisture, all working in harmony, if normal, towards mastication and insalivation for the well being of all the rest of the tract and its dependents. In no other part of this thirty-two feet is there a place so equipped with the necessities for mastication and insalivation, and in no part of this tract is there a place that functionates well without this previous mastication and insalivation. With these first functions impaired there will be faulty digestion; with imperfect digestion there will follow incomplete assimulation; with impaired assimulation will be impaired metabolism; with impaired metabolism will be impaired health, and fruitless is a life without health.

In the days of the colonies it was clearly felt that attacks upon Massachusetts or Virginia were not only attacks upon those states, but were a menace to all the colonies; that their past struggles, their future hopes, and all the experiences of the past and the promises of the future had been the means of weaving the fibres of government into such a strong fabric that no section of the fabric could be mis-used except at the expense of the whole. Similarly it is more true today that a serious injury to the mouth is not only an attack upon the mouth and the rest of the digestive tract, but upon the entire body. Therefore, it behooves us to fortify well this strategic point, for nowhere in the body is there a place where the proper heat, moisture and food supply unite so favorably for the development and growth of germ life as in the mouth. Here a great number of species have been found to exist; many of which are disease producing, including such as are productive of tuberculosis, pneumonia, typhoid, diptheria, dental caries, etc.

Enormous sums of money are being spent to purify the air we breathe, the food we eat, and the water we drink, and such expenditures no fair thinking mind will criticise; but we should know that all these precautions are inefficient so long as the mouth is a garbage receptacle, contaminated with millions of disease producing microbes with which the pure food, the pure water and the pure air must come in contact and so become as impure, if not more so—than at first, before they reach the fields of usefullness for which they were purified. The mind of the public is in the right attitude toward the influence of the mouth, but they are lacking in the proper method of approach to the subject and it becomes our duty to teach them the only true way of solving this problem as it confronts them.

They recognize the danger and avoid the use of the public communion cup, the public drinking cup and the spitting in public places. This is all good and should be adhered to closely, but it deals only with the effect without teaching the cause, and we cannot scientifically and intelligently deal with any question without first dealing with the cause, and the cause of these ill-effects is certainly a defective mouth, the special field of the dentist and the field through which good health and civilization can be greatly advanced.

Never in the history of scientific research toward prevention and cure of human ills has so much attention been given to specializing on the many fields. Not many years ago the general practitioner of medicine was called upon to care for all the diseases to which human beings were heir, but now we are living in this good age when such conditions are history and we are able to have all the organs that greatly influence health cared for skillfully by competent men who have through choice, rather than of necessity, chosen their special fields of usefulness.

We all believe that to have good health that every organ having a function in preserving life must receive intelligent care; certainly the mouth has an important function, as a very useful and necessary function is contributed by it in obtaining and maintaining the proper nourishment for the preservation of life. We readily concede the necessity of specialists for eye diseases, specialists for skin diseases, specialists for stomach diseases, etc., who should be highly skilled in knowledge of function, pathology and treatment, and be able to give the public sufficient knowledge that they may care for themselves in a way that will reduce to a minimum the necessity of assistance by the physician. I am sure then if it is beneficial that we have men to specialize in the treatment of these parts when diseased, it should be more necessary to have the dental profession take its stand in the circle of preventive medicine and assist the public in guarding the common point of attack, the mouth, and thereby more completely eliminate disease and the necessity of the other specialists whose fields are primarily dependent upon the mouth.

So important are all the teeth to the welfare of the body that not one temporary tooth should be lost until nature's time, as a premature loss causes a failure of the bone at that point to properly develop, resulting in a crowded condition of the premanent set, giving improper relations between opposing and approximating teeth, making mastication but a pretense, and at the same time predisposing the gums to disease by placing an excessive amount of work on some and allowing others to go idle. (You will note just here that both underwork and overwork are predisposing factors towards disease conditions of the soft tissues in the mouth.) Too early loss of the temporary teeth may not only mean a loss of their usefulness, the usefulness of the permanent set and the usefulness of the soft tissues, but such neglect allowed to go uncared for, may have marked influence over the character, life and destiny of the individual. We find when the teeth are sacrificed through neglect and not through nature's plan, that both jaws may suffer in deformity as a result. We find also that when the soft tissues, upon which the bones are in a degree relying for sustenance, are diseased and poorly nourished, that the bone tissue likewise suffers to the degree of its dependence and therefore fails its full development.

Of the twenty-two bones in the head, the upper jaw articulates with nine, the lower jaw with one, the upper has twelve pair of muscles attached to it, the lower has fifteen. Hence when one of these bones fails in full development, the influnce of such is manifest directly upon ten bones and twenty-seven pairs of muscles and indirectly upon every bone and muscle in the head. If then, the Cranium, the home of the brain, is influenced in its development, who will question but that the brain shares equally in such a misfortune. The brain is the center of the nervous system and under the direction of this great central station, the home office, the brain, the nervous system goes forth throughout the body as master over all the other systems, and when this master fails to direct the affairs of the body it it clear that the subordinate organs will not do their full duty as the laws of health demand, and a diseased body it the result, which indeed, should be avoided at all times. This is, however, by no means the climax of this degenerating influence as the body of flesh is not only the dwelling place of man proper, but is the higher home of the very image of God, and no man is doing his full duty to himself, his country and his God who is not holding this ideal up as his chief concern of his life, and the one who best provides for the character of man is the one who understands best the value of the house in which we live, the body, and no one understands this better and is striving to preserve it more perfectly than the dentist. If then, the dentist can influence the Cranium, and thereby the brain, he then is a conspicuous factor in deciding the destiny of an individual, for the brain is the seat of the mind and as one thinks, so is he, and from what it is springs all his acts, and from his acts spring his habits, and from his habits spring his character and from his character comes his destiny.

Therefore, I am sanguine in the belief, that if the mouth is properly cared for throughout the developing age, the inmates of our infirmaries, asylums, prisons, etc., will be surprisingly decreased, as such inmates are so often results of debauched characters in childhood. As evidence of such a theory it is noted that such inmates show neglected mouths in childhood in a marked degree over the average, while the examination of business and professional men, or the men who think and push the world, it is found that their mouths have had more professional attention during childhood than the masses. Then, childhood, being the time when the physical and mental attributes are either made are marred, the dentist should be the chief of all councils that contribute to these physical and mental attributes, for of the 1,500,000 people who die yearly in the United States forty per cent. of their diseases, or 600,000, are preventable and 500,000 of this number should have been prevented through the dentist's efforts, giving us the opportunity of being the mightiest force at work in life-saving. Let us then so use this waiting opportunity that we shall force the world to give honor to him to whom honor is due, for he who is instrumental in preserving a human body is serving also the human soul and does most, therefore, not only for time, but for eternity.

But you may say these are all very nice facts and theories, but our profession is one of limited prestige and powerless to demonstrate these facts and theories, and I grant that we are weak in numbers, but the power remains with us to be strong in influence, for the strength of any cause is determined by that cause itself; as has been well said, the "humblest citizen in all our land, when clothed in the armour of a righteous cause, has greater power in his conflict for humanity than all the combined forces of intellect struggling in behalf of a cause of error." Thus I have endeavored to present a few facts in behalf of a cause of truth, a cause that may not be supported by the combined intellectual powers, but a cause as just as any ever solved by a thinking humanity —the cause of dentistry—its place and influence in modern civilization. These facts are not new to the profession but are new to the public and here it is that we are falling short of our duty by not seizing this opportunity to serve humanity by making ourselves teachers of the cause of dentistry whenever the occasion is ours, and the occasion is ours with each patient that is ours. To deny that the public mind is not in accord with such an attitude, would be to deny the facts of history where similar moves have been pressed to perfection through the aid of the public. Already we can hear the tread of the advance guard of the public pressing close behind us. So we *must* advance with the onward march of progress to save our reputations, the valor and the honor of our profession—and so entitle ourselves to the degree of "Doctor of Dental Surgery."

The public has already taken a most commendable stand for the betterment of home, religion and state, thereby developing those fibres in human lives that are absolutely essential for the production of proud and noble characters. Without such public support no advocate of an onward march in any of the walks of life could obtain favorable results for we are now in an age when all individuals, callings or professions are interdependent. So the dental profession in recognition of this fact as we stand on the threshold of a new era, the era of preventive science, should stand here with outstretched arms of welcome, imploring the assistance in the intelligent and scientific care of the most used and the most abused, the most needed and the most mistreated of all the organs of nature's handiwork—the human mouth. Through it may be seen beauty to behold or unsightliness untold, according to the care or neglect that has been given it. Through it may come financial wealth through health, or financial unease through disease according to the care or neglect that has been given it.

Knowing then these things as we do, it becomes inhumane and next to criminal for us to see those who are dependent upon us for counsel and guidance, suffering from a neglect of our duty to our chosen profession, for unquestionably we can have marked influence, as I have endeavored to show, over the health, the life and the destiny of an individual when cared for in the proper way at the proper time. We are truly our brother's keeper and this realization is the crying need of the dentist today, and when once realized we shall reap as our reward an advanecd civilization over the present. Then shall we know from a task well done that there are "Tongues in the trees, books in the running brooks, sermons in the stones and good in everything."

### DISCUSSION.

Dr. W. E. Lundy: This paper was to have been discussed by a much older soldier in this warfare than I have the honor to be, Dr. J. L. Mewborn, but since he is not with us, there are a few things I would like to emphazize.

Dr. McRae has covered the ground, I think, pretty thoroughly, so far as the larger per cent. of our profession is concerned, but he also speaks of the masses needing education. Personally, I believe there is a great lack of education in this particular of the dentists themselves.

We accept a great many of the statements he has made in a general way, like a man believing in God, and not being a Christian, he accepts Christianity, but he don't accept enough of it to save him, and it is just about that way with the dental situation at present. We believe these things to be true, but we don't let anyone else realize how much they really need them, or how much truth there is in them. A dentist comes to his office in the morning, and pretty soon after in comes some mother with two or three little folks that she has had to bundle up in order to get there with the one that is in trouble, and you look over the mouths and find—what? The first thing you find is the sixth-year molars decayed. You state to her that some of the first molars are permanent teeth, and she will proceed to tell you immediately that he has never shed these teeth. Somebody should have told that woman long ago the fact that the child cut these permanent teeth at six years of age. She has several other children that have been to the dentists and their teeth have been filled. There is an illustration that we have neglected to educate while we work.

Dr. Towner once said something about me that at the time I did not take as a big compliment. He said some could tell more than they knew, but he said Lundy is another man who will not tell as much as he knows. I don't know whether I am now telling more than I know or not, but I am trying to tell every patient that comes in some things that he ought to know, and don't know, and I am trying to tell it in such a way that he will believe it, whether it be false or true. We should try to live up to this sort of teaching. If you do not think the patient is listening, all you have to do is just to put out a few bad statements, and see how soon you will hear from him.

The Doctor has emphasized what great influence the mouth has over a man's future life and his future state, health, comfort, and even his moral character and hope of the Great Beyond, etc., just how much of that is true we shall probably never know. You let a man have something that is eternally irritating him, and he is just as unfit for the duties of life as it is possible for him to be. If you will pardon me for the expression, let him have a corn, and have to stand on it all day, and you will find that he will give you a short answer for something that if he was normal would not irritate him at all. The trouble begins in the early part of life, a person begins to lose some of his temporary teeth, and then he loses a little here and a little more somewhere else, and as the doctor pictured it, he goes from one step to another. The loss of a single tooth interferes with the digestion, and a little later that interferes with something else. The idea is if we expect to have a perfect individual, we must get in our work before the mischief is done. We all know that a large proportion of the mouths we come in contact with are ruined beyond the possibility of restoring them to a perfect mouth before we ever get hold of them.

We are right on the threshold of an era of dental appreciation. The Dental Hygiene Association is sowing this land with good and wholesome literature. The plans are well laid, and the field is ripe, certainly. The laborers are ready to work, they are ready for battle, and they are going right into it. The public is actually asking for these things before we give them to them, and it is up to us to get busy.

I believe I heard from Dr. Cottrell there is to be a little exposition in Knoxville. I don't know the nature of it exactly, but it is to occupy possibly a month, and they have asked for both the oral hygiene and oral prophylaxis. The prophylactic idea has spread in that section of the country from somebody, I don't know who has put it out—might have come largely from the Ladies Home Journal, for all I know, and it shows, as the Doctor says in his paper, that they are right on our heels, and we must be up and doing if we are to seize the opportunity we have been clamoring for so long. As a matter of fact, I do not think the medical men are losing any sleep over it, as they have all they can look after.

I am told that a number of prominent physicians here in this city had a case in the hospital in which a man had been unconscious the better part of the time for several weeks, and they had not been able to locate what was the matter with him. They did not know. He was just going down all the time. Finally they called a dentist in consultation, and they found that man's mouth just reeking with Pyorrhea. There wasn't a medical man present that had taken note of that fact, so far as we know. The public takes notice of this sort of thing. I don't want to say a word against the medical practitioner, for heaven knows he has more piled on him than any one man has any business with, but that just shows that they are leaving that field absolutely alone, leaving us to look after it, and if we do not educate the public, we can not blame the general practitioner for it.

Dr. H. A. Holder: The first criticism I would make on the paper is that the Doctor starts off with an apology. I do not think that paper needs any apology at all. It was beautifully written, beautifully worded, and starts right down at the cradle and carries a fellow right on up through the whole subject. I don't think it needs any apology at all. I want to thank the Doctor for the paper and the suggestions he has thrown out in it.

Possibly a great many of us haven't realized before just the influence that these things the Doctor has spoken of have—what the loss of one or two of the temporary teeth, the influence they might have upon the development of the child, the physical development and the moral and mental development of the child.

In the experiments that were carried out in Cleveland a few years ago, and of which we all read in the journals it was demonstrated very clearly there that the efficiency of school-children can be increased 100 per cent. in a great many cases simply by putting their mouths in a hygienic condition. If this be a fact, and we know it is, because it has been proven beyond the shadow of a doubt, it certainly behooves us to get busy in our own communities, get busy among our own patients, and spread this gospel, of oral hygiene and show the people what can be done. They are beginning to realize what can be done, and as Dr. Lundy said, Dr. Cottrell who was talking to us this morning, and was telling us that the people are coming to the dentists there, and calling for the oral hygiene exhibit there in that exposition. The Dentists there did not go and say, "We want to put in an oral hygiene exhibit," but the managers came to the dentists and asked them for it. They know it is a good thing, and they want the people to be educated along that line. Let us educate ourselves. Let us get to work, and work together on this question. If we do, there is no telling what good we will accomplish for the benefit of humanity, and of course fit ourselves in a certain measure to reap some of the benefits also.

Dr. W. J. Morrison: I have been a member of this Association about twenty-five years, and I think the first time I ever attended a meeting of the Association this subject was discussed. I think it was at Jackson, Tenn., and some phase of it has been discussed at every meeting from that time to this. I just say this to show you how slow the public are to take hold of these important matters. I want to say, also, it shows the necessity of untiring perseverance. It shows the necessity of overcoming obstacles, such as that met in Nashville, about three years ago, when we invited the Mayor and City Council and City Officials up to Carnegie Library, and gave them an exhibit of the pictures we would like to show to the school children, and to deliver lectures on oral hygiene in the schools. I wasn't on the Committee, but we were met with this discouraging attitude of the Board of Education; they very seriously objected to our proposition on the ground that we were trying to advertise somebody, they said it was a commercial enterprise, and we wanted to make business for the dentists. I think our Secretary here can give some experience that he has had along this line in trying to educate the public by a concerted effort of the dentists.*

But with all that, gentlemen, with all the draw-backs and all the obstacles that we have met, and all that we might meet, we do not want to become discouraged, because I think that every man who has taken an interest in the matter has seen a wonderful progress along this line. We can now pick up almost any journal, or newspaper, or magazine, and find an article on oral hygiene, where formerly all you ever saw in print on oral hygiene and the practice of dentistry was some joke about somebody that went in to have his teeth extracted, and fill perhaps a column and a half, as I saw in the newspaper not a great while ago, about a man that went in to have his teeth extracted, or filled, and I think there was about one and a half columns of the agony he underwent in having that operation performed.

The reason I speak is that there are so many young men here that have not been keeping up with this department of this subject, that I want to tell them they must not be discouraged, but must keep right on.

It is a grand thing to have the men of the Knoxville Exposition come to the dentists. We have had to go to them all the time before and on bended knees, and beg them to allow us to teach the children the care of the teeth and now, we find that they have dental offices in the schools in some cities, and the children's teeth are examined, and those who are not able to pay for having their teeth attended to receive attention free, and those who are able to pay are given a chart to take home to their parents, showing the defects in the mouth, and they can go to their own dentists, and have them looked after. Think of what progress that is in twenty-five years! Twenty-five years ago there wasn't a school in the United States that had a dental office attached to it. Now-a-days, not only that, but they are carrying it a little further. In some of the great manufacturing institutions you can not get a position until you produce a certificate from a reputable dentist that your teeth and mouth are in good condition. Of course this is one of the modern ways that the manufacturers have adopted—selfish, it is true—of securing efficiency. Suppose the engineer should have to leave his work—the whole factory stops. You see they have realized the necessity, from a commercial point of view, of every man keeping his oral cavity in good condition for efficiency sake, what ought we not do for healths sake.

Dr. Murray McRea: I was in hopes that there would be some more discussion on my paper, as I have enjoyed the discussions and criticisms that have been made, and if there is any merit at all in the paper I ask that it be attributed not so much to myself, as to the influence of the Memphis Dental Society, because it is from my association with that body, that I have gathered most of the facts put forth in the paper and the inspiration to write it.

One gentleman asked me a while ago the object of my paper and I was very glad the subject of the paper was printed in the program, so he would know what it was I was writing about. The subject was given out before the paper was written, and the object was to get together ourselves, so that we might understand each other better, that we might better grasp the importance of getting our own selves to understand just what we are here for.

